# Supplementary figures and images for: Whole-brain radiation therapy plus simultaneous integrated boost for brain metastases from breast cancers
Source: PeerJ. 2024 Jul 12;12:e17696. doi: 10.7717/peerj.17696 (PMC11248998; doi:10.7717/peerj.17696)

# Mean brain dose

+ High

+ Low

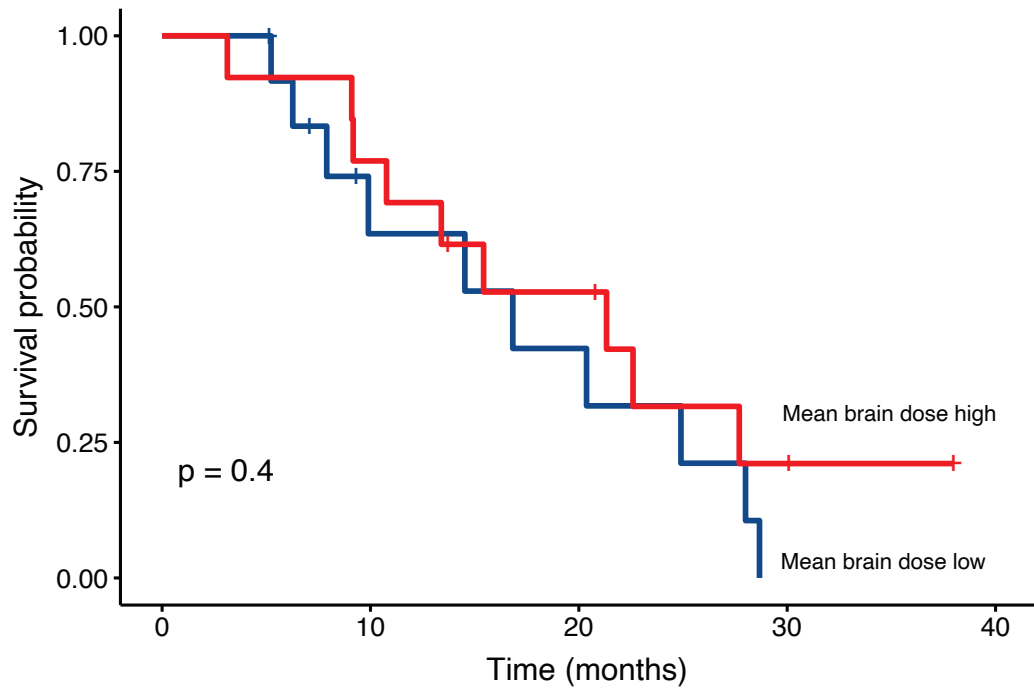

Supplement: Supplemental Information 2 [file peerj-12-17696-s002.pdf]
