# Supplementary material for: Whole-brain radiation therapy plus simultaneous integrated boost for brain metastases from breast cancers
Source: PeerJ. 2024 Jul 12;12:e17696. doi: 10.7717/peerj.17696 (PMC11248998; doi:10.7717/peerj.17696)
Supplement: Supplemental Information 3 [file peerj-12-17696-s003.docx]

**Supplementary table 1 Dose constrain of organs at risk**

| OARs | Brain stem | Spinal cord | Optic nerves | Chiasm | Lens | Eyes |
| --- | --- | --- | --- | --- | --- | --- |
| D_max_ (Gy) | 54 | 45 | 50 | 50 | 9 | 50 |

Abbreviations: OARs, organs at risk; D_max_, maximum dose.
