# Supplementary material for: Whole-brain radiation therapy plus simultaneous integrated boost for brain metastases from breast cancers
Source: PeerJ. 2024 Jul 12;12:e17696. doi: 10.7717/peerj.17696 (PMC11248998; doi:10.7717/peerj.17696)
Supplement: Supplemental Information 4 [file peerj-12-17696-s004.docx]

**Supplementary table 2 Comparison of radiation volumes and doses**

|  | Overall | Complete response | Partial response | Not evaluated | P |
| --- | --- | --- | --- | --- | --- |
|  | n=27 | n=4 | n=14 | n=9 |  |
| Volume of GTV (cm^3^) | 8.80 (3.15-20.90) | 4.45 (2.58-6.10) | 9.10 (5.40-17.75) | 22.60 (2.30-34.70) | 0.155 |
| D_max_ of GTV (cGy) | 4963.80 (4915.58-5039.60) | 5040.40 (4431.80-5253.00) | 4948.30 (4915.58-5018.20) | 4993.30 (4926.40-5087.10) | 0.562 |
| D_mean_ of brain (cGy) | 3513.50 (3455.22-3592.72) | 3506.50 (3317.80-3927.20) | 3497.85 (3440.62-3527.02) | 3519.40 (3512.20-3621.70) | 0.535 |

All values were expressed as median (IQR). Abbreviations: D_max_, maximum dos; D_mean_, mean dose; GTV, gross tumor volume.
